# Indonesian nurses’ awareness and application of reasonable adjustments when caring for people with intellectual disability and/or autism

**DOI:** 10.1111/inr.12959

**Published:** 2024-03-20

**Authors:** Amy Pracilio, Nathan J. Wilson, Roxsana Devi Tumanggor, Andrew Cashin

**Affiliations:** ^1^ Faculty of Health and Human Sciences Southern Cross University Lismore New South Wales Australia; ^2^ School of Nursing and Midwifery Western Sydney University Richmond New South Wales Australia; ^3^ Faculty of Nursing University of Sumatera Utara Medan Indonesia; ^4^ Faculty of Health and Human Sciences and Health Clinic Southern Cross University Lismore New South Wales Australia

**Keywords:** Autism, curriculum, intellectual disability, nursing, professional development, reasonable adjustments

## Abstract

**Aim:**

This study aimed to understand Indonesian nurses’ familiarity with the concept of reasonable adjustments, and the frequency of its application within their practice.

**Background:**

People with intellectual disability and/or autism are exposed to significant barriers when accessing healthcare and have poorer health outcomes than those without developmental disabilities. Reasonable adjustments can improve accessibility and quality of healthcare for people with disabilities and involves adapting practices and environments to promote equitable healthcare.

**Introduction:**

There is a scarcity of literature focused on the application of reasonable adjustments in the Indonesian nursing context. A greater understanding of the application of reasonable adjustments in this context can help inform nursing curricula and policy.

**Methods:**

A cross‐sectional, descriptive survey ‐was undertaken and is reported in accordance with the Strengthening the Reporting of Evaluations and Non‐randomised Designs. Following descriptive analysis, bivariate analyses were undertaken between key demographic, workplace, and self‐reported capability variables, and familiarity and implementation of reasonable adjustments.

**Results:**

The majority of respondents were not familiar with the concept of reasonable adjustments and self‐reported sometimes applying it within their practice. Higher levels of educational and clinical exposure to intellectual disability and/or autism, and self‐capability variables, were significantly associated with familiarity with reasonable adjustments and their application.

**Conclusions:**

An increase in nursing curricula focused on caring for people with intellectual disability and/or autism, including content focused on applying reasonable adjustments, is indicated.

**Implications for nursing policy:**

Given that, internationally, people with intellectual disability and/or autism have disproportionately negative health outcomes and experiences, findings highlighting gaps in understanding and application of reasonable adjustments of Indonesian nurses have substantial implications for nursing policy and curriculum.

## BACKGROUND

People with intellectual disability (ID) and/or autism spectrum disorder (ASD) are exposed to significant barriers when accessing healthcare (Poole et al., 2022). Despite international legislation and frameworks instilling the rights of people with disabilities to equal and accessible healthcare (United Nations, 2006), people with ID and/or ASD experience poorer health outcomes than those without developmental disorders. People with ASD are less likely to receive primary care interventions and have higher rates of chronic illness and mortality (Cashin et al., 2018), and people with ID experience greater levels of chronic and complex health conditions, higher rates of hospitalisation, and greater post‐care complications (Iacono et al., 2020). These disparately negative outcomes are compounded by environmental and psychosocial barriers that impact the ability of people with ID and/or ASD to access adequate healthcare (Doherty et al., 2020; Walsh et al., 2020). Additionally, mainstream healthcare environments often do not consider, and are not designed for the cognitive, sensory, and communication needs of people with ID and/or ASD (Giarelli et al., 2014; Howie et al., 2021a).

The implementation of reasonable adjustments to health settings and practices is a key pillar in removing barriers and improving healthcare outcomes and experiences for people with ID and/or ASD. For example, under The Equality Act (2010) in the United Kingdom, all public health services (including hospitals) are required to apply reasonable adjustments for people with disability using that service. Reasonable adjustments involve making changes to organisational, social, and physical environments to promote equitable access to healthcare (Kersten et al., 2023; Moloney et al., 2021). They can be applied instantaneously at the individual patient level in response to the care needs of the person with ID and/or ASD, as well as through the organisational and procedural levels that anticipate healthcare needs (Heslop et al., 2019; Marsden & Giles, 2017). Adaptions to the healthcare environment can include organisational changes as well as modifications to the physical environment to reduce stressors and sensory stimuli and improve accessibility (Kersten et al., 2023; Moloney et al., 2021). The environmental needs of people with ASD can be addressed by the reduction of sensory overload, including the modification of intense lighting and overstimulating sounds (Howie et al., 2021a). People with ID can often experience multimorbidity and have comorbid visual, hearing, and physical impairments that require adjustments to the navigation of healthcare environments including wheelchair accessibility and modified wayfinding information (Howie et al., 2021a; Kersten et al., 2023). Additionally, procedural adjustments such as modified waiting times, appointment options, and discharge procedures can improve healthcare experiences. Reasonable adjustments to social environments largely focus on adapting communicative approaches for people with ID and/or ASD, including people with verbal and non‐verbal communicative needs. Utilising health passports, including caregivers in conversations, social stories, and communication aids are key examples of social adjustments (Marsden & Giles, 2017).

Nursing is theoretically underpinned by notions of holistic, person‐centred care. Thus, nurses are well placed to implement reasonable adjustments when caring for people with ID and/or ASD. However, several studies have noted a deficit in the nursing educational curriculum focused on caring for these cohorts (Furst & Salvador‐Carulla et al., 2019; Trollor et al., 2018). In addition to low levels of education, the literature unequivocally highlights nurses reporting feelings of unpreparedness, lack of confidence, and communication barriers when caring for people with ID and/or ASD (Appelgren et al., 2022; Howie et al., 2021b; Khanlou et al., 2023; Lewis et al., 2017).

Nurses’ use of reasonable adjustments when caring for people with ID and/or ASD has been explored within the literature but is largely concentrated on single contexts, especially acute settings. Studies focused on applying reasonable adjustments for people with disabilities more broadly (Heslop et al., 2019), as well as those focused on people with ID (MacArthur et al., 2015), have explored the positive role of a disability liaison nurse while also noting a lack of knowledge of ID, and communication issues as barriers to adjusting practice experienced by less specialised nurses (Redley et al., 2019). Outside of the United Kingdom, Australia, and the Republic of Ireland, specialised nursing roles such as disability liaison nurses are rare, so such findings are not necessarily internationally transferable. A number of single‐site studies focused on nurses applying reasonable adjustments for people with ASD have explored the types of adjustments that prove most helpful including person‐centred care, use of modified language tools, and sensory modifications to sounds and light (Lalive d'Epinay Raemy & Paignon, 2019; Moyle & James et al., 2015). A key limitation to the current body of literature is that studies are largely focused on Europe and the United Kingdom and are mainly concentrated on single nursing contexts, rather than comparative studies of diverse nursing settings.

The largest identified study, by sample size, focused on nursing and reasonable adjustments was an Australian study of 693 Registered Nurses (RNs) across varied contexts of practice (Wilson et al. (2022). This study was one part of a larger 2020 survey of RNs’ educational exposure to ID/ASD content, and self‐reported levels of knowledge, confidence, comfort, and preparedness to care for these cohorts (Cashin et al., 2021). Key findings highlighted that Australian RNs reported relatively low levels of familiarity with the concept of reasonable adjustments, when compared to those who applied them in their nursing practice (Wilson et al., 2022). Additionally, nurses who reported higher levels of comfort, confidence, and knowledge were more likely to be familiar with the concept and regularly use reasonable adjustments in their practice. This current study aims to fill a key gap in international nursing literature through the replication of this survey in the Indonesian nursing context.

Over the last decade, Indonesia has increased the promotion of the rights of people with disabilities, including those with developmental disabilities. As of 2017, it was estimated that 4%–11% of the Indonesian population have some form of disability (Cameron & Contreras Suarez, 2017). As a signatory to the UN *Convention on the Rights of People with Disabilities* (2006), Indonesia has enacted a number of national laws protecting freedom from discrimination, and equal access to health, education, political, and employment rights (Mayor of Bandung, 2018; President of the Republic of Indonesia, 2011). These laws protect the rights of people with physical, intellectual, mental, and long‐term sensory disabilities. Although equal and accessible healthcare for people with ID and/or ASD is enshrined in law, the reality of its application and whether healthcare professionals utilise reasonable adjustments has not been subjected to a research study. Additionally, although the Indonesian Nursing Act of 2014 noted that nursing care must include people with ‘special needs’, presently only 5% of curriculum content is focused on care for people with disabilities (Association of Indonesian Nurse Education Institution, 2016). In 2023, the Indonesian Health Act (Ministry of Health, 2023) was introduced, and while disability was identified, no specific reference was made to people with ID and/or ASD.

There is no published literature focused on the experiences of Indonesian nurses applying reasonable adjustments when caring for people with ID and/or ASD. Several small, single‐context studies have, however, focused on challenges experienced by Indonesian nurses when caring for these cohorts. For instance, a phenomenological study by Erwati and Keliat (2018) highlighted the communicative difficulties experienced by nursing students when caring for people with ID, including an inability to communicate with colleagues about their patients with ID. Additionally, in a phenomenological study of 11 nurses and early identification and intervention for children with developmental disabilities, Mardiyanti et al. (2020) noted that Indonesian nurses saw their role as focused on general nursing, curative management, and monitoring delay rather than providing systematic intervention which differed from international standards. A greater exploration of Indonesian nurses’ understanding and application of reasonable adjustments will help inform Indonesian nursing curricula development and aid cross‐cultural comparison.

## METHODS

### Research aim

To understand and describe Indonesian nurses’ self‐reported familiarity with the concept of reasonable adjustments, and its implementation within their practice when caring for people with ID and/or ASD in mainstream health settings. Additionally, associations between key demographic, workplace, and self‐reported capability variables and levels of familiarity and implementation of reasonable adjustments were explored.

### Survey design and rigour

The broader study utilised an exploratory, cross‐sectional design and a descriptive survey instrument to explore Indonesian nurses’ self‐reported educational and clinical preparedness, knowledge comfort, and confidence to care for people with ID and/or ASD. The survey instrument was translated and adapted from a survey of Australian RNs conducted in 2020 (Cashin et al., 2021). The original instrument was translated from English to Bahasa Indonesian and piloted with 30 nurses from a university hospital in Medan, Indonesia. The creators of the Australian survey were consulted prior to this pilot to ensure meaning was not lost. The pilot process focused on content validity, comprehension, and the transferability of items to the Indonesian nursing context. Three items specific to the Australian context were removed: Indigenous status, registration with the Australian nursing authority, and membership to a professional college or organisation. Seven items were modified to better reflect the Indonesian context, including registration status, membership with an Indonesian nurses’ organisation, gender, governance status and context of nursing practice, and highest level of tertiary education. These items were altered to better reflect the structure and conceptualisation of demographic information (including gender) and the context of nursing education and organisational structures in Indonesia.

The final survey instrument included 32 items across seven main domains. Domain 1 focused on demographic information and included nine questions, whilst the second domain included six questions about the current nursing role and context of practice. Domains 3–7 focused on self‐reported knowledge and competency to provide care to people with ID and/or ASD. These domains covered the following themes: educational and clinical exposure to content relevant to people with ID and/or ASD (three questions), knowledge (one question), preparedness (two questions), confidence (two questions), comfort (eight questions), and making reasonable adjustments (three questions).

This study focuses on the domain about making reasonable adjustments and their association with key demographic, workplace, and self‐reported capability variables. Questions asked respondents about their familiarity with the concept of reasonable adjustments (yes/no) and frequency of intentionally undertaking reasonable adjustments in their practice (often/sometimes/never). For respondents who reported ‘sometimes’ or ‘often’ undertaking reasonable adjustments, they were asked to choose which categories of adjustments they intentionally undertook. The following options were presented to respondents, and they could choose multiple responses: (1) adapting nursing approach; (2) adapting discharge options; (3) adjusting workload/team's skillset; (4) allowing more time for communication and/or physical disabilities affecting time management; (5) check for stimuli that can trigger behaviours that challenge; (6) communicate patient's ID/ASD to a colleague verbally; and (7) seek out adjustments in the setting.

### Ethical considerations

The study was approved by the Medical and Health Research Ethics Committee at Gadjah Mada University, Indonesia (Approval Reference: KE/FK/1191/EC/2022). All participants taking part in an online seminar were given the opportunity to participate in the survey and were assured that the choice not to participate did not affect their ability to take part in the learning opportunity.

### Participants and data collection

The main inclusion criterion was to be a nurse in Indonesia. The survey was conducted in September 2022 following an online seminar focused on the management and early detection of ID and ASD. Data were collected on Google Forms, using an anonymous link with implied consent sent to all attendees of the seminar. Attendees were from diverse regions of Indonesia and varied nursing contexts.

### Data analysis

Data were translated by the third author from Bahasa Indonesian to English in Microsoft Excel, prior to data cleaning, and categorisation using IBM SPSS Statistics (Version 28). Descriptive and inferential data analyses were undertaken using SPSS.

Descriptive statistics are presented as frequencies (*n*), percentages (%), and where relevant as mean and standard deviations. Analyses with Chi‐squares and where appropriate Fisher's exact were utilised for inferential statistics. The key dependent variables were (1) familiarity with the concept of reasonable adjustments and (2) frequency of intentionally undertaking reasonable adjustments in nursing practice. The independent demographic and workplace variables tested were (1) age, (2) gender, (3) type of degree, and (4) immediate family member with ID and/or ASD. Certain demographic variables, such as gender, were investigated due to differences in nursing behaviour highlighted in previous studies between male and female nurses (Juanamasta et al., 2021). Whilst having an immediate family member with ID and/or ASD was chosen to explore whether there was an association between this and understanding/application of reasonable adjustments. Key independent variables relevant to education and experience caring for people with ID and/or ASD were (1) ID and/ or ASD undergraduate content; (2) ID and/or ASD clinical placement; (3) ID and/or ASD Continuing Professional Development; (4) educational preparedness; (5) clinical preparedness; (6) confidence addressing healthcare needs; (7) confidence to refer onto appropriate services; (8) knowledge about comorbidities/healthcare needs of people with ID and/or ASD; and (9) all eight domains focused on comfort levels performing tasks relevant to caring for people with ID and/or ASD.

## RESULTS

### Demographic profile and reasonable adjustments

The final sample included 544 completed responses, reflecting an 89.7% response rate. Overall, 566 survey responses were received with 41 attendees of the seminar not consenting to participate and 20 responses were not eligible as they were undergraduate students. The gender distribution was 415 females (76.3%) and 129 males (23.7%), with a mean age of 34.8 years old (SD = 7.65). Almost all respondents (*n*; %) were born in Indonesia (542; 99.6) and completed their pre‐nursing registration education there (543; 99.8). The majority were from the Batak ethnic group (204; 37.5), followed by Jawa (182; 33.5).

The Diploma of Nursing was the highest level of education for the majority of respondents (247; 45.4), followed by Bachelor of Nursing and a one‐year internship (198; 36.4), Master's degree (48; 8.8), Bachelor of Nursing (47; 8.6), Master's degree and nursing specialisation (3; 0.6), and Doctorate (1; 0.2). Over two‐thirds of respondents were employed by the government sector (364; 66.9), in comparison to a private institution (180; 33.1). The majority of respondents worked in a hospital setting (375; 68.9), followed by public health centre (82; 15.1) and university (28; 5.1). Additionally, almost all were not currently in a specialist ID and/or ASD nursing role (533; 98.0).

Over half of respondents reported they were not familiar with the concept of reasonable adjustments (323; 59.4%). Whilst the majority of respondents reported ‘sometimes’ (282; 51.8%) or ‘never’ (228; 41.9%) intentionally applying reasonable adjustments in their practice. For those that ‘often’ (34; 6.3%) or ‘sometimes’ applied reasonable adjustments, the types of adjustments they made are illustrated in Appendix A. Of note, more than two‐thirds reported adapting their nursing approach as the most common form of reasonable adjustments undertaken (220; 69.6), followed by seeking out adjustments in the setting (103; 32.5), and allowing time for communication and/or physical disabilities affecting time management (86; 27.2) (see Appendix A).

### Demographic and workplace characteristics and reasonable adjustments

Analyses were undertaken to identify any associations between demographic and workplace variables and the two dependent reasonable adjustment variables (see Table 1). Respondents with immediate family members with ID and/or ASD were significantly more likely to be familiar with the concept. Male nurses were significantly more likely to ‘sometimes’ make reasonable adjustments, whereas female nurses were significantly more likely to report ‘never’ making reasonable adjustments. Additionally, nurses reporting that they ‘never’ make reasonable adjustments were more likely to be slightly older than those reporting ‘often’ undertaking them.

**TABLE 1:** Demographic and workplace profile by reasonable adjustment variables.

	Familiarity with concept of reasonable adjustments				Regularity making reasonable adjustments				
Variable Level	Yes (*n *= 221; 40.6%)	No (*n *= 323; 59.4%)	Test statistic	*p*	Often (*n = *34; 6.3%)	Sometimes (*n *= 282; 51.8 %)	Never (*n *= 228; 41.9%)	Test statistic	*p*
Age, mean (SD)	33.64 (7.4)	35.71 (7.7)	*t *= −126		33.0 (7.1)	33.89 (7.4)	36.36 (7.7)	ANOVA	<0.001
Sex,									
Male	57 (44.2)	72 (55.8)	*χ* ^2^(1) = 0.889	0.346	4 (3.1)	80 (62.0)	45 (34.9)	*χ* ^2^(2) = 8.05	0.018
Female	164 (39.5)	251 (60.5)			30 (7.2)	202 (48.7)	183 (44.1)		
Governance status									
Private institution	78 (43.3)	102 (56.7)	*χ* ^2^(1) = 0.818	0.366	7 (3.9)	111 (61.7)	62 (34.4)	*χ* ^2^(2) = 10.99	0.004
Government	143 (39.3)	221 (60.7)			27 (7.4)	171 (47.0)	166 (45.6)		
Highest level of education									
Diploma prepared	97 (39.3)	150 (60.7)	*χ* ^2^(2) = 0.352	0.839	13 (5.3)	141 (57.1)	93 (37.7)	*F *= 5.34	0.249
Bachelors prepared	102 (41.6)	143 (58.4)			18 (7.3)	117 (47.8)	110 (44.9)		
Postgraduate degree	22 (42.3)	30 (57.7)			3 (5.8)	24 (46.2)	25 (48.1)		
Immediate family members with ID/ASD									
Yes	41 (59.4)	28 (40.6)	*χ* ^2^(1) = 11.57	<0.001	6 (8.7)	41 (59.4)	22 (31.9)	*χ* ^2^(2) = 3.52	0.166
No	180 (37.9)	295 (62.1)			28 (5.9)	241 (50.7)	206 (43.4)		

### Education and reasonable adjustments

Exposure to educational content and clinical placement relevant to caring for people with ID and/or ASD was significantly associated with familiarity and implementation of reasonable adjustments in nursing practice (see Table 2). Nurses who did not have ID and/or ASD content in their undergraduate curriculum or experienced a clinical placement relevant to caring for these cohorts were significantly less likely to be familiar with the concept of reasonable adjustments. This pattern was also evident in regard to Continuing Professional Development (CPD), where nurses not exposed to CPD focused on ID and/or ASD were significantly less likely to be familiar with reasonable adjustments.

**TABLE 2:** Education, knowledge, confidence, and reasonable adjustments.

	Familiarity with concept of reasonable adjustments				Regularity making reasonable adjustments				
Variable Level	Yes (*n *= 221; 40.6%)	No (*n *= 323; 59.4%)	Test statistic	*p*	Often (*n = *34; 6.3%)	Sometimes (*n *= 282; 51.8 %)	Never (*n *= 228; 41.9%)	Test statistic	*p*
ID/ASD content									
Yes	130 (49.4)	113 (50.6)	*χ* ^2^(1) = 16.36	<0.001	23 (8.7)	143 (54.4)	97 (36.9)	*χ* ^2^(2) = 8.77	0.012
No	91 (32.4)	190 (67.6)			11 (3.9)	139 (49.5)	131 (46.6)		
Clinical placement									
Yes	57 (62.0)	35 (38.0)	*χ* ^2^(1) = 20.88	<0.001	11 (12.0)	55 (59.8)	26 (28.3)	*χ* ^2^(2) = 12.0	0.002
No	164 (36.3)	288 (63.7)			23 (5.1)	277 (50.2)	202 (44.7)		
CPD ID/ASD									
Yes	26 (68.4)	12 (31.6)	*χ* ^2^(1) = 13.08	<0.001	4 (10.5)	27 (71.1)	7 (18.4)	*F = *10.39	0.004
No	195 (38.5)	311 (61.5)			30 (5.9)	255 (50.4)	221 (43.7)		
Preparedness—Education									
Very	39 (69.6)	17 (30.4)	*χ* ^2^(2) = 55.0	<0.001	15 (26.8)	34 (60.7)	7 (12.5)	*χ* ^2^(2) = 40.55	<0.001
Somewhat	143 (47.4)	159 (52.6)			18 (6.0)	182 (60.3)	102 (3.7)		
Not at All	39 (21.0)	147 (79.0)			9 (4.8)	66 (35.5)	111 (59.7)		
Preparedness—Clinical									
Very	34 (72.3)	13 (27.7)	*χ* ^2^(2) = 68.7	<0.001	7 (14.9)	28 (59.6)	12 (25.5)	*χ* ^2^(4) = 52.6	<0.001
Somewhat	148 (50.2)	147 (49.8)			19 (6.4)	183 (62.0)	93 (31.5)		
Not at All	39 (19.3)	163 (80.7)			8 (4.0)	71 (35.1)	123 (60.9)		
Confidence addressing health needs									
Very	27 (62.8)	16 (37.2)	*χ* ^2^(2) = 64.8	<0.001	6 (14.0)	20 (46.5)	17 (39.5)	*F = *62.24	<0.001
Somewhat	178 (48.6)	188 (51.4)			24 (6.6)	225 (61.5)	117 (32.0)		
Not at All	16 (11.9)	119 (88.1)			4 (3.0)	37 (27.4)	94 (69.6)		
Confidence to refer on									
Very	30 (71.4)	12 (28.6)	*χ* ^2^(2) = 66.9	<0.001	5 (11.9)	23 (54.8)	14 (33.3)	*F = *46.29	<0.001
Somewhat	175 (47.2)	196 (52.8)			25 (6.7)	220 (59.3)	126 (34.0)		
Not at All	16 (12.2)	115 (87.8)			4 (3.1)	39 (29.8)	88 (67.2)		
Knowledge about comorbidities/healthcare									
Very	24 (75.0)	8 (25.0)	*χ* ^2^(2) = 66.9	<0.001	5 (15.6)	16 (50.0)	11 (34.4))	*F = *63.49	<0.001
Somewhat	164 (50.0)	164 (50.0)			25 (7.6)	205 (62.5)	98 (29.9)		
Not at All	33 (17.9)	151 (82.1)			4 (2.2)	61 (33.2)	119 (64.7)		

Lack of educational and clinical exposure to ID and/or ASD was also significantly associated with the frequency of intentionally applying reasonable adjustments in practice. Nurses reporting no undergraduate content, clinical placement, or CPD were also significantly more likely to report ‘never’ implementing reasonable adjustments (see Table 2).

### Preparedness, confidence, knowledge, and reasonable adjustments

Significant associations were identified between levels of preparedness, confidence, knowledge, and familiarity with reasonable adjustments (see Table 2). Of note, nurses who reported that their undergraduate education and clinical experience did “not at all” prepare them to care for people with ID and/or ASD were also significantly more likely to report a lack of familiarity with the concept of reasonable adjustments. Additionally, nurses reporting feeling ‘not at all’ knowledgeable about the healthcare needs and comorbidities of people with ID and/or ASD, and ‘not at all’ confident in their ability to address these needs or refer to appropriate services, were significantly less likely to know about reasonable adjustments. This pattern was reversed for nurses who reported feeling ‘very’ knowledgeable, prepared, and confident. Across all variables, they were significantly more likely to be familiar with the concept of reasonable adjustments. Additionally, nurses reporting low levels of preparedness, confidence, and knowledge were significantly more likely to “never” intentionally implement reasonable adjustments (see Table 2).

### Comfort and reasonable adjustments

**FIGURE 1:**
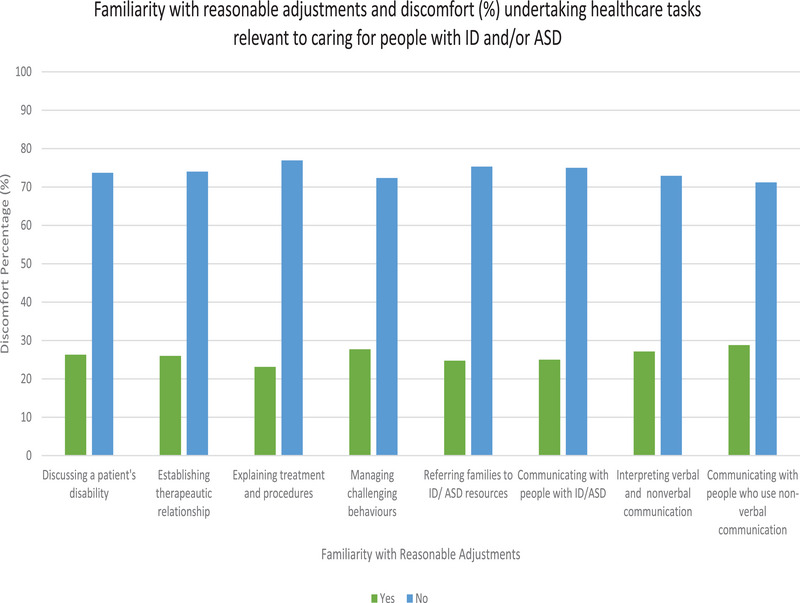
Familiarity with reasonable adjustments and discomfort levels.

Analyses were undertaken to determine if there was an association between the two reasonable adjustment variables, and all eight comfort domains (Appendix B). Significant associations were found between familiarity with the concept of reasonable adjustments, and comfort levels with undertaking tasks relevant to caring for people with ID and/or ASD across all eight domains. Of note, nurses who reported lower levels of comfort (not comfortable at all/slightly comfortable) were significantly less likely to be familiar with the concept of reasonable adjustments. Highly significant relationships were identified between low levels of comfort explaining treatment or procedures, referring families to ID/ASD resources, communicating with people with ID/ASD, and interpreting verbal and non‐verbal communication, and lack of familiarity with reasonable adjustments. Figure 1 illustrates this relationship between higher levels of discomfort undertaking tasks related to ID and/or ASD care and low levels of familiarity with reasonable adjustments. No significant associations were identified between levels of comfort and frequency of applying reasonable adjustments (see Figure 1).

## DISCUSSION

This is the first study with a large sample about reasonable adjustments in the Indonesian context. In the Australian study that utilised the same survey (Wilson et al., 2022), it was identified that while the majority of respondents were not familiar with the concept of reasonable adjustments, the majority of respondents reported making adjustments sometimes or always. The majority of the participants in the current study were also not familiar with the concept of reasonable adjustments; however, in contrast to the Australian sample, the majority reported adjusting practice sometimes or never. This difference may be related to the fact that person‐centred care does not underpin the Indonesian nursing philosophy as it does in Australia (Cashin et al., 2017). In line with the Australian sample (Wilson et al., 2022), a significant relationship was identified between preregistration educational content related to ID and/or ASD and having a clinical placement with a focus on ID and/or ASD and familiarity with the concept of reasonable adjustments and feelings of knowledge, preparedness and comfort making adjustments to practice. This identified relationship suggests that content relevant to caring for people with ID and/or ASD has an association with key knowledge relevant to their care, including reasonable adjustments. In contrast to the Australian sample, where there was not a significant relationship identified between self‐identified knowledge of reasonable adjustments and self‐report of making adjustments, in the current sample those with reported knowledge of reasonable adjustments were significantly more likely to report making adjustments.

Although male participants were not significantly more aware of the concept of reasonable adjustments, they were significantly more likely to sometimes make adjustments, as opposed to females who were more likely to never make adjustments. This may reflect gender difference in the practice of nursing in Indonesia, where males have historically been afforded more role autonomy (Juanamasta et al., 2021). This difference is noteworthy as in Indonesia, based on an estimate of all health workers, it is estimated that 30% of Indonesian nurses are male (Efendi et al, 2022). A slightly lower portion of 23.7% participated in the current sample.

There has been a significant established link between exposure to curricula content and ongoing education related to ID and/or ASD and self‐reported confidence, comfort, and preparedness to provide nursing care to people with ID and/or ASD in general (Cashin et al., 2021) and specifically in relation to making reasonable adjustments (Wilson et al., 2022). This link between education and practice elements is important, particularly in relation to the identified low level of curricula content in pre‐registration/licensure nursing courses internationally (Appelgren et al., 2018; Desroches et al., 2019; Trollor et al., 2018). The identified small proportion of related content in Indonesian nursing courses (Association of Indonesian Nurse Education Institution, 2016) could plausibly have a direct relationship with the low self‐reported confidence, comfort, and preparedness to make reasonable adjustments and the frequency with which practice is adjusted to meet the needs of people with ID and/or ASD. This significant relationship was also noted between low levels of CPD relevant to caring for people with ID and/or ASD and being more likely to “never” apply reasonable adjustments, implying resources could also focus on strengthening professional development options for Indonesian nurses improving skills in this area. As the 2023 Indonesia Health Act (Ministry of Health, 2023) for health professionals does not refer to people with ID and/or ASD, there is a chance that the dedicated proportion of content may diminish further as curricula focus on national health priorities, when the findings of the current study indicate the need for an increase.

### Implications for nursing and health policy

Utilising reasonable adjustments in nursing care for people with ID and/or ASD is a key strategy to help improve healthcare quality, outcomes, and experiences, as well as reduce barriers to accessibility (Moloney et al., 2021). Internationally, a greater understanding of gaps in nurses’ awareness and application of reasonable adjustments is imperative to ensure curricula and policy are focused on rectifying gaps in knowledge and practice.

### Limitations

The findings are based on self‐report. The findings may be skewed by the participants having an interest in learning about ID and/or ASD as attending CPD in this domain.

## CONCLUSION

An increase in curricula content that explicitly includes content related to adjusting practice is indicated. Ideally, all students will experience a dedicated clinical placement in caring for people with ID and/or ASD. Further research is indicated that moves beyond self‐report and is based on audit or observation.

## AUTHOR CONTRIBUTIONS

*Study design*: AP, NW, RT, AC. *Data collection*: RT. *Data analysis*: AP, NW, AC. *Study supervision*: NW, RT, AC. *Manuscript writing*: AP, NW, RT, AC. *Critical revisions for important intellectual comment*: AP, NW, RT, AC.

## CONFLICT OF INTEREST STATEMENT

No conflict of interest has been declared by the author(s).

## ETHICS STATEMENT

The study was approved by the Medical and Health Research Ethics Committee at Gadjah Mada University, Indonesia (Approval Reference: KE/FK/1191/EC/2022).

## Supporting information

Supporting Information

Supporting Information
