# Supplementary material for: Indonesian nurses’ awareness and application of reasonable adjustments when caring for people with intellectual disability and/or autism
Source: Int Nurs Rev. 2024 Mar 20;71(4):1005–14. doi: 10.1111/inr.12959 (PMC11600539; doi:10.1111/inr.12959)
Supplement: Supplementary file 2 — Supporting Information [file INR-71-1005-s001.docx]

| Variable | | | Familiarity with concept of reasonable adjustments | | Test  statistic | *p* | Regularity making reasonable adjustments | | | | | Test  statistic | | *p* | |
| --- | --- | --- | --- | --- | --- | --- | --- | --- | --- | --- | --- | --- | --- | --- | --- |
|  | Level | | Yes  (*n*=221; 40.6%) | No  (*n*=323;  59.4%) |  |  |  | Often (*n=*34; 6.3%) | Sometimes (*n*=282;  51.8 %) | Never (*n*=228; 41.9%) |  | |  | |  |
| **Discussing patient’s disability with other health professionals** | | |  |  |  |  |  |  |  |  |  | |  | |  |
|  | Very Comfortable/Comfortable  Not at All/Slightly Comfortable | | 201 (42.9)  20 (26.3) | 267 (57.1)  56 (73.7) | *χ*^2^(1)= 7.499 | .006 |  | 31 (6.6) 3 (3.9) | 244 (52.1) 38 (50.0) | 193 (41.2) 35 (46.1) | *F=*.965 | | .620 | |  |
| **Establishing therapeutic relationship** | | |  |  |  |  |  |  |  |  |  | |  | |  |
|  | Very Comfortable/Comfortable  Not at All/Slightly Comfortable | | 202 (42.9) 19 (26.0) | 269 (57.1)  54 (74.0) | *χ*^2^(1)=7.488 | .006 |  | 32 (6.8) 2 (3.7) | 243 (51.6) 39 (53.4) | 196 (41.6) 32 (43.8) | *F=*1.569 | | .491 | |  |
| **Explaining treatment or procedure** | | |  |  |  |  |  |  |  |  |  | |  | |  |
|  | Very Comfortable/Comfortable  Not at All/Slightly Comfortable | | 203 (43.6) 18 (23.1) | 263 (56.4) 60 (76.9) | *χ*^2^(1)= 11.624 | <0.001 |  | 31 (6.7) 3 (3.8) | 245 (52.6) 37 (47.4) | 190 (40.8) 38 (48.7) | *F=*1.956 | | .392 | |  |
| **Managing/de-escalating challenging behaviours** | | |  |  |  |  |  |  |  |  |  | |  | |  |
|  | Very Comfortable/Comfortable | | 193 (43.6) | 250 (56.4) | *χ*^2^(1)= 8.560 | .003 |  | 30 (6.8) | 233 (52.6) | 180 (40.6) | *F=*2.049 | | .383 | |  |
|  | Not at All/Slightly Comfortable | | 28 (27.7) | 73 (72.3) |  |  |  | 4 (4.0) | 49 (48.5) | 48 (47.5) |  | |  | |  |
| **Referring families to ID/ASD resources** | | |  |  |  |  |  |  |  |  |  | |  | |  |
|  | | Very Comfortable/Comfortable | 198 (43.9) | 253 (56.1) | *χ*^2^(1)= 11.748 | <0.001 |  | 30 (6.7) | 236 (52.3) | 185 (41.0) | *F=*1.137 | | .579 | |  |
|  | | Not at All/Slightly Comfortable | 23 (24.7) | 70 (75.3) |  |  |  | 4 (4.3) | 46 (49.5) | 43 (46.2) |  | |  | |  |
| **Communicating with people with ID/ASD** | | |  |  |  |  |  |  |  |  |  | |  | |  |
|  | | Very Comfortable/Comfortable | 194 (44.5) | 242 (55.5) | *χ*^2^(1)= 13.639 | <0.001 |  | 27 (6.2) | 231 (53.0) | 178 (40.8) | *χ*^2^(2)=1.184 | | .553 | |  |
|  | | Not at All/Slightly Comfortable | 27 (25.0) | 81 (75.0) |  |  |  | 7 (6.5) | 51 (47.2) | 50 (46.3) |  | |  | |  |
| **Interpreting verbal and nonverbal communication** | | |  |  |  |  |  |  |  |  |  | |  | |  |
|  | | Very Comfortable/Comfortable | 189 (44.4) | 237 (55.6) | *χ*^2^(1)=11.396 | <0.001 |  | 26 (6.1) | 229 (53.8) | 171 (40.1) | *χ*^2^(2)=2.930 | | .231 | |  |
|  | | Not at All/Slightly Comfortable | 32 (27.1) | 86 (72.9) |  |  |  | 8 (6.8) | 53 (44.9) | 57 (48.3) |  | |  | |  |
| **Communicating with people who use nonverbal communication** | | |  |  |  |  |  |  |  |  |  | |  | |  |
|  | | Very Comfortable/Comfortable | 187 (43.9) | 239 (56.1) | *χ*^2^(1)=8.715 | .003 |  | 25 (5.9) | 228 (53.5) | 173 (40.6) | *χ*^2^(2)=2.324 | | .313 | |  |
|  | | Not at All/Slightly Comfortable | 34 (28.8) | 84 (71.2) |  |  |  | 9 (5.6) | 54 (45.8) | 55 (46.6) |  | |  | |  |
